# Subjects of Social Welfare
‡"Subjects of Social Welfare." By the Right Hon. Sir Lyon Playfair, K.C.B., M.P., LL.D., F.R.S. (Cassell and Co.)


**Published:** 1890-02-08

**Authors:** 


### Subjects of Social Welfare.‡

Speeches and addresses are at first sight likely to be considered as of only ephemeral interest, devoted as they are to special subjects and to special audiences. Presumably, they are intended to enlighten a particular section of the great public on some particular topic of temporary interest. Magazine articles, again, are of somewhat similar nature, with this difference, that the section of the public to which they are addressed is wider and often more intelligent. But the underlying notion that they are ephemeral is more or less true of them all, and generally speaking they are relegated to oblivion and lose their individual reputation after contributing their quota to the making of history. There are, however, some subjects of chronic interest, which periodically become prominent and of ephemeral importance. As instances we may quote vaccination, vivisection, disposal of the dead, protection, marriage with a deceased wife's sister, and so on. Without being in any sense questions for political parties, they are from their very nature perpetually recurring and perpetually being debated on the platform and in the Press. It is extremely unlikely that they will ever be finally disposed of, and hence it is well that the spoken and written opinions of individuals, whose authority and right to a hearing are undisputed, should be rescued from the files of newspapers and magazines and reprinted in book form, and so made more easily accessible for future reference. Sir Lyon Playfair has thus dealt with several of his contributions to these periodical discussions, and has republished them under the apt and comprehensive title, "Subjects of Social Welfare." The volume is divided into three sections, treating respectively of Public Health, Industrial Wealth, and National Education. It includes addresses to the Social Science Association, the Royal Institution, Manchester, the National Liberal Club, speeches delivered in Parliament and to his constituents at Leeds, and contributions to various magazines. By laymen as well as by professional political economists this book is likely to be cordially received. Sir Lyon Playfair's language is remarkably plain and free from technical mannerisms, his arguments are lucid, and his facts well marshalled. He wastes no time in useless verbiage, but goes straight to the point he intends to demonstrate or controvert, and with all this never sins by being dull or tedious. Owing probably to the fact that the volume is a collection of speeches and articles produced on separate occasions, and not a book written, so to speak, at one time, there is a certain amount of over-lapping and reiteration. This is especially noticeable in the paper on the "Displacement of Labour by Modern Inventions," published originally in the *Contemporary Review*, and an address on "Industrial Competition and Commercial Freedom," delivered to the National Liberal Club in 1888, and which in this volume come immediately next to one another. The speech in defence of vivisection is a concise résumé of the principal arguments for and against, ably elucidated, and, as we think, convincingly established. The reply to the resolution moved by Mr. Taylor in 1883 on behalf of the Anti-vaccination Association is of special interest again now, in the light of the further development of the question as set forth in Mr. Crookshank's recent book. Perhaps the most able portion of the volume is that devoted to industrial wealth. The four articles on "Depression of Agriculture and Fair Trade," "The Displacement of Labour by Modern Inventions," "Industrial Competition and Commercial Freedom," and "The Effect of Protection on Wages" are particularly powerful, although, as we have said, it is here that the fault of overlapping is most apparent —if under the circumstances it be a fault. The third, and last section, treating of national education, is of high interest and of increasing importance in these days when competition is passing from material to mental and intellectual regions. Sir Lyon Playfair's book will form a useful addition to the library of everyone who takes a practical interest in the social problems of the day. He propounds his views simply, and if we disagree with some of his conclusions we must admire his methods of reasoning. Convenient in size, and agreeably printed, "Subjects of Social Welfare" contains a mine of information that many will find pleasure in exploring.

